# Prolapse of the Bladder in a Case of Labor

**Published:** 1880-11

**Authors:** H. D. Valin

**Affiliations:** 800 S. Halsted Street; Chicago


### ARTICLE VII.

PROLAPSE OF THE BLADDER IN A CASE OF LABOR. By H. D. VALIN, M.D., Chicago. (Reported at a meeting of the West Chicago Medical Society, Oct. 25th.)

On October 23d, at 4 p.m., I was called to attend Mary B. in her seventh confinement. She was at the eighth month, and labor was brought on by over-work. A first examination revealed a pediculated tumor projecting between the labia and out of the vulva. The anterior wall of the vagina could be traced from the meatus urinarius around the tumor to the anterior lip of the os uteri, which could be easily reached, and was dragged downward and toward the symphysis pubis. A fold of the tumor pressed between the fingers was a few lines in thickness, being composed of the fundus of the bladder, and the anterior wall of the vagina, and could not be mistaken for the amniotic membranes.

The prolapsed bladder contained two or three ounces of urine which could be pressed back above the pubis, and which disappeared after micturition. This tumor after being nearly replaced carried the anterior lip of the os uteri about an inch higher up.

This was dilated enough to admit the ends of two fingers, and remained so for five hours; there being no regular labor pains. She was then administered one drachm of fluid extract of ergot, and after an hour and a half, the uterus was contracting regularly and strongly. When the head passed the inferior strait, it pushed a fold of the bladder down between itself and the symphisis pubis, causing intense suffering to the mother. The head was small and soon escaped under the pubis, without any interference.

The mother had been subject to incontinence of urine for some months previous, and complained of unusual pain in the lumbar region during this labor which lasted seven hours.

Such cases are rare. Mme. Lachapelle mentions one, and Merriman reported one in which the bladder was punctured, but a slight degree of prolapse is said to be common in women who have borne large families.

*Diagnosis.* The anatomical attachment of the bladder, the thickness of its walls, the urine disappearing on micturition or catheterizing, and the ease in replacing that viscus, help the diagnosis. But it can not be mistaken for the bag of waters, particularly if the latter can be felt within the os uteri during a pain, which do not affect the bladder to any extent.

*Treatment.* Leishman recommends to evacuate the bladder, to replace the fundus, holding it in position until the head descends; this is obviously difficult. Cazeaux advises to hold the bladder in position with a catheter while the head is born; the catheter should be soft and flexible for fear of doing serious injury to the urethra.

800 S. HALSTED STREET.

---

The following lines are by Benserade, and thus rendered by Dr. Johnson:

In bed we laugh, in bed we cry;
And born in bed, in bed we die.
The near approach, a bed may show,
Of human bliss to human woe.

